# Design of the offline test electronics for the nozzle system of proton therapy

**DOI:** 10.1038/s41598-024-62515-z

**Published:** 2024-05-27

**Authors:** Peng Huang, Zhiguo Yin, Tianjian Bian, Shigang Hou, Fengping Guan, Shizhong An, Yang Wang, Tianjue Zhang, Luyu Ji, Lipeng Wen, Xueer Mu

**Affiliations:** China Institute of Atomic Energy, Beijing, 102413 China

**Keywords:** Proton therapy, Ionization chamber, Position signal, Beam fluence signal, Signal simulation, Electrical and electronic engineering, Engineering, Radiotherapy

## Abstract

A set of nozzle equipment for proton therapy is currently under development at China Institute of Atomic Energy (CIAE). To facilitate the off-line commissioning of the whole equipment, a set of ionization chamber signal generation system, known as the test electronics, was designed. The results showed that the system can simulate the beam position, beam fluence (which exhibits a positive correlation with the dose), and other related analog signals generated by the proton beam when it traverses the ionization chamber. Moreover, the accuracy of the simulated beam position is within ± 0.33 mm, and the accuracy of the simulated beam fluence signal is within ± 1%. The test electronics can output analog signals representing environmental parameters. The test electronics meets the design requirements, which can be used for the commissioning of the nozzle system as well as the treatment control system without the presence of the proton beam.

## Introduction

A proton therapy system based on the 230 MeV superconducting cyclotron is currently under development at CIAE^1–4^. The nozzle system, which mainly consists of three ion chambers and two scanning magnets, plays an essential role in the process of Pencil Beam Scanning (PBS). In proton therapy, in order to irradiate tumor lesions according to the preset position and dose (represented as the charge collected by the ionization chamber), precise control of the beam scanning process by the PBS system is essential^5–7^. During spot-scanning irradiation, the ionization chamber (IC) monitors the position and fluence of the beam in real time. When the target dose reaches the set value, the beam is turned off and the next spot is irradiated, and so on. If the position/dose error of the beam exceeds the predefined error threshold, the interlock is triggered to turn off the beam. Currently, the dose accuracy of the final irradiation of proton therapy devices typically ranges from ± 2 to ± 3%, whereas the position accuracy generally ranges from ± 0.5 to ± 1.5 mm^8–11^.

**Figure 1:**
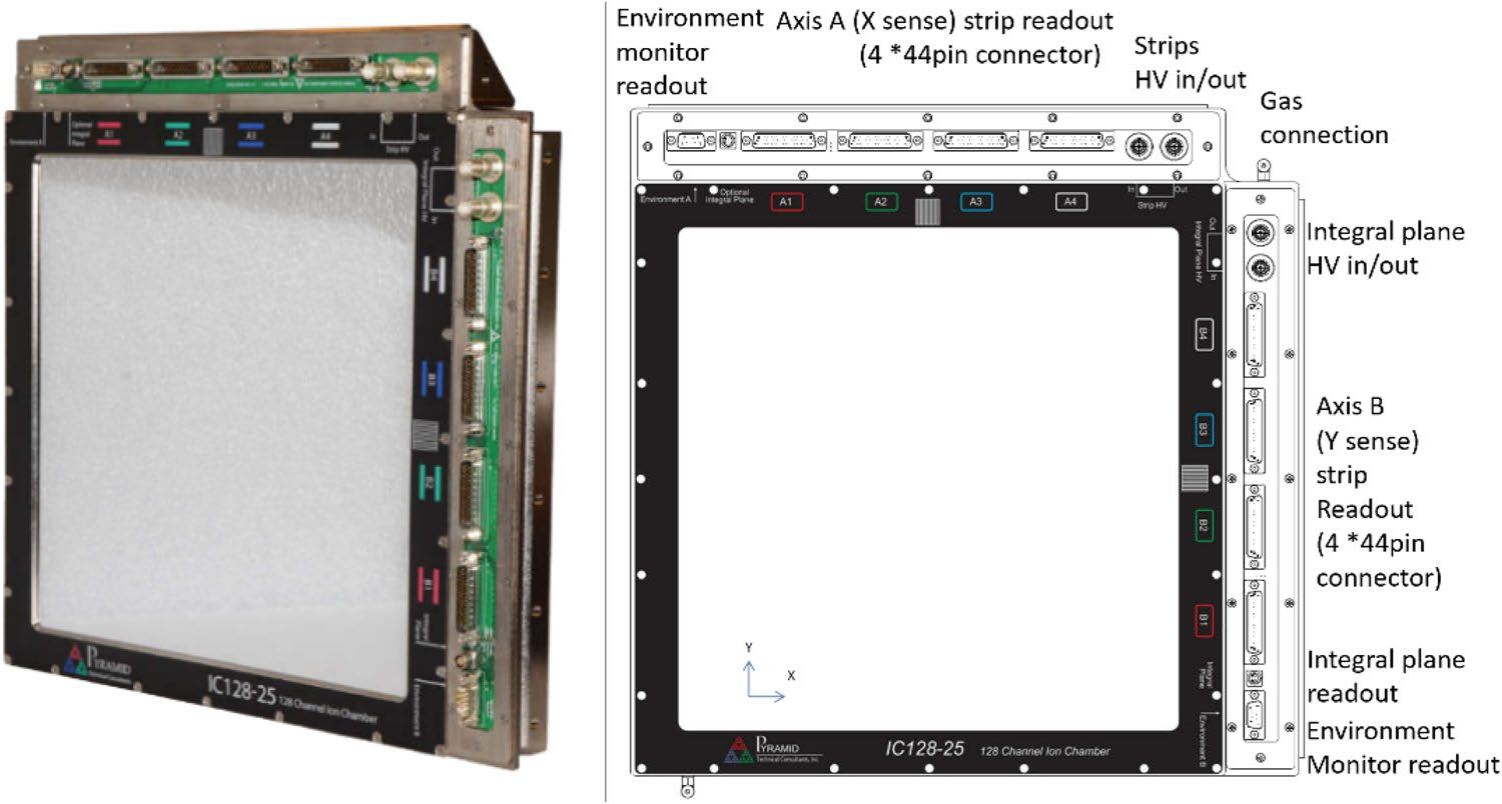
Structure of the parallel plate ionization chamber.

**Figure 2:**
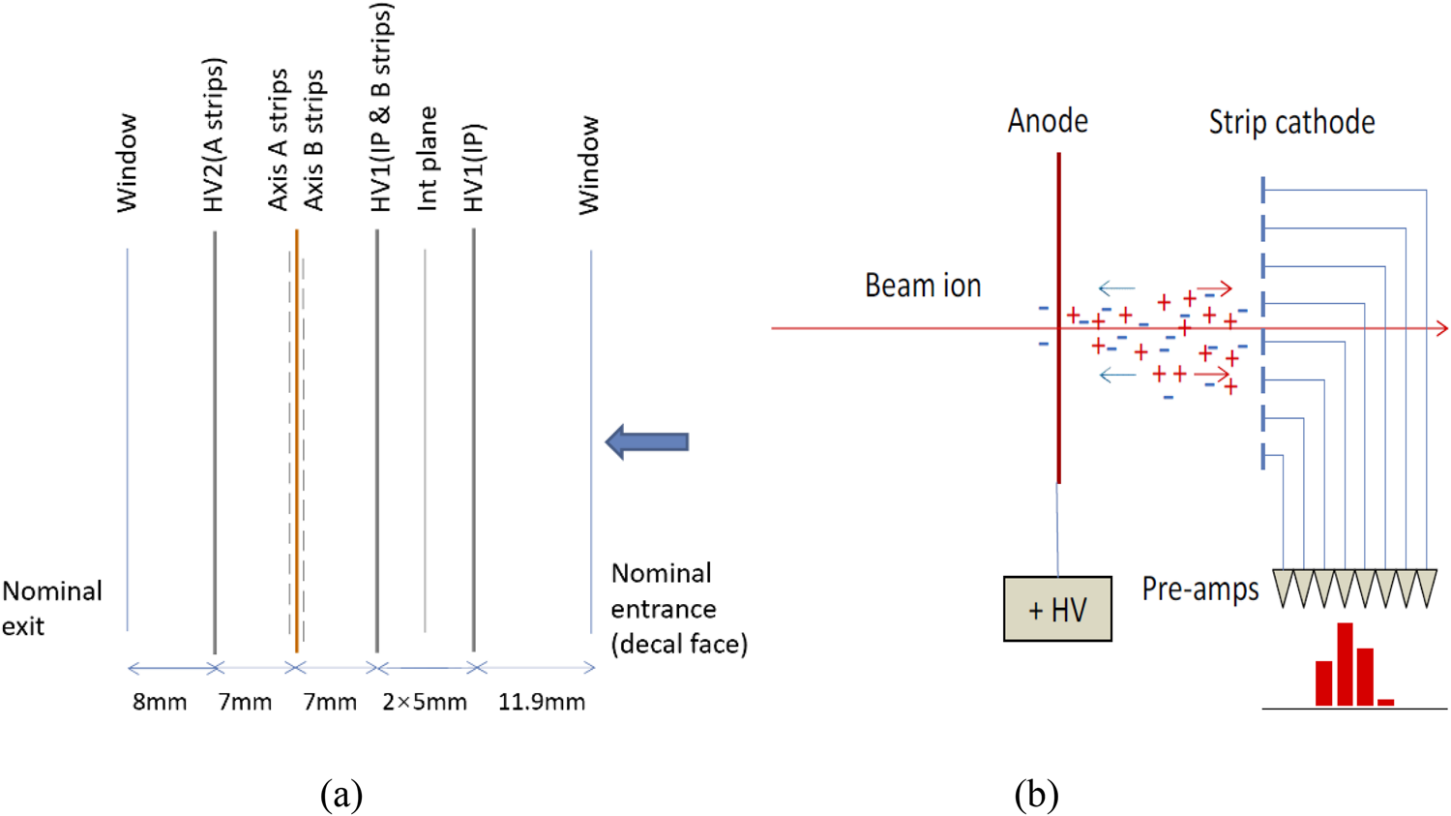
(**a**) Sequence of electrode foils and (**b**) signal partition on strip cathodes.

Figure 1 shows the structure of the parallel plate ionization chamber^12^, in which the strip layer (Axis A strips or Axis B strips) is used to collect the beam position signal, and there are 128 strips for each direction. The ionization chamber has two directions X/Y, resulting in 256 strips in total. The width of a single strip is 1.89 mm, and the gap between two adjacent strips is 0.11 mm, resulting in an overall spacing of 2 mm between strips. The integral plane (Int plane) is used to collect the ionization charge which is correlated with the dose delivered by the beam. The high voltage layers (HV1 and HV2) are used to supply a 2 kV positive bias voltage. The ionization chamber is equipped with temperature and pressure sensors, which are used to transmit environmental information through the Environment monitor readout port to the front-end electronics for the calibration of the readout data. The integral plane electrode collects charge from the whole active area of the IC and delivers it to a single readout channel. The strip electrodes partition the measured charge according to where it is formed across the readout area. The partitioning is linear and direct because the field is uniform in the gap. The induced signal and charge eventually arriving at the cathodes is routed by the electric field onto the strips. Figure 2 shows the sequence of electrode foils and the signal partition on strip cathodes.

The beam information is required to facilitate testing and upgrades of the on-site nozzle system and its electronics. In addition, the beam-related signals are required for the operation of the treatment control system (TCS)^13,14^, otherwise, the treatment process cannot be conducted. Therefore, in order to complete the commissioning of the nozzle system and the TCS system, a set of offline test electronics was designed to simulate the signals generated by the IC. This approach eliminates the dependence on the beam. Once completed, the system can be used to debug and upgrade the entire treatment facility anytime, and at any location, independent of the beam quality and accelerator operating status.

## Theoretical design of the test electronics

**Figure 3:**
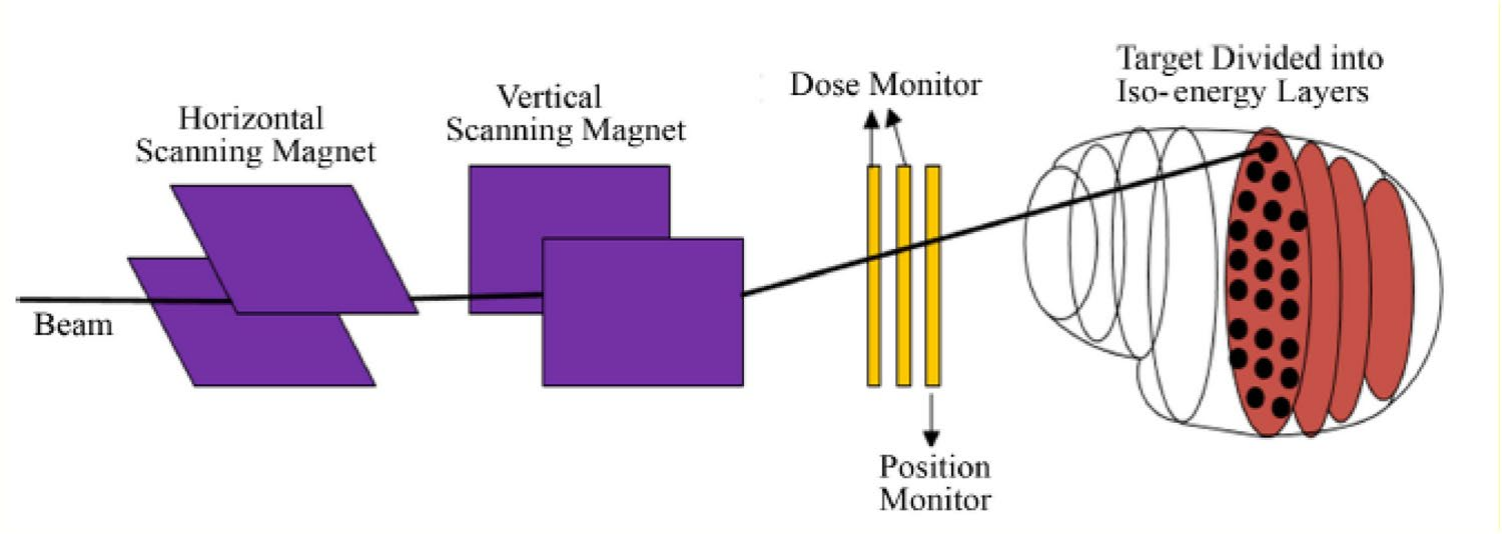
Schematic of pencil beam scanning system.

As shown in Fig. 3, the typical PBS nozzle system^15–17^ consists in different subsystems allowing to control and monitor the beam^18^. The beam with different energies has different irradiation depths, and the radiation depth increases with the increase of the energy. Typically, one iso-energy layer may contain dozens or even more scanning spots. The scanning magnets are used to control the beam position and the ion chambers are used to monitor the beam position and the irradiation dose in realtime^19–21^. The beam is turned on at the scanning spots and turned off between scanning spots, and that is the so called spot-scanning irradiation mode adopted by our cyclotron-based proton therapy facility. The other two pencil beam scanning modes are the raster scanning and the line scanning modes. The raster-scanning mode delivers particals when the beam is moved from one spot to another, whereas the line-scanning provides a continuous dose delivery. For the offline test system, it is also designed to emulate the spot-scanning mode.

**Figure 4:**
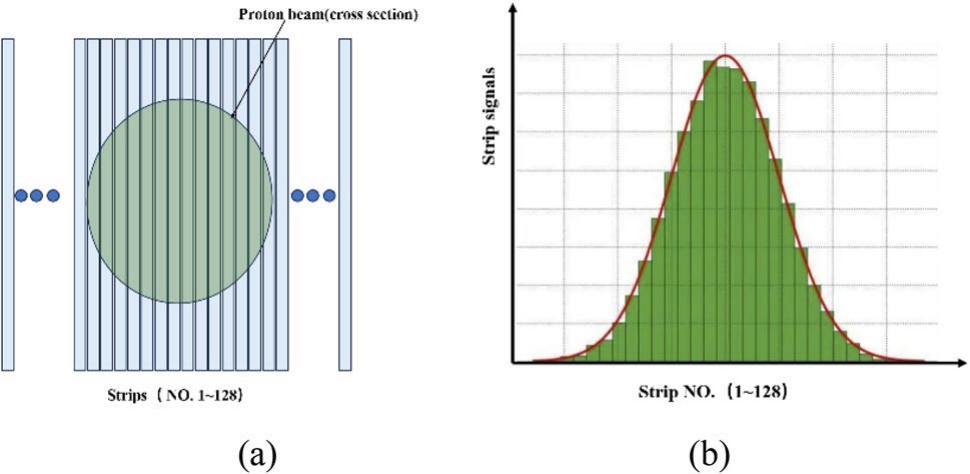
Beam signals with Gaussian distribution.

Since the beam itself exhibits Gaussian distribution^22,23^, the signals detected by the strips also exhibit Gaussian distribution. The size of the beam spot (also expressed as beam sigma) determines the range (or number) of the strips through which the beam passes. For example, if the beam sigma is 4 mm, then there may be 12 strips (24 mm range) that will generate current signals according to the 3 Sigma rule, and the strip pitch is 2 mm. Furthermore, the intensity of the beam current determines the amplitude of signals collected on the strips. In addition, the position coordinates of the beam determine on which strips the signals can be collected. Figure 4a shows the schematic diagram of the beam passing through the ionization chamber strips (X direction), and Fig. 4b shows the signals generated on these strips, adhering to the Gaussian distribution outlined in Formula (1) below:

1 $$f(x)=\frac{1}{\sqrt{2\pi }\sigma }\text{exp}\left(-\frac{(x-\mu {)}^{2}}{2{\sigma }^{2}}\right),$$

where, μ represents the center position of the beam, σ denotes the standard deviation. The beam position is in the irradiation field (30 cm × 40 cm), and the beam size (σ) is about 3.2–4.4 mm for 70–230 MeV proton beam.

**Figure 5:**
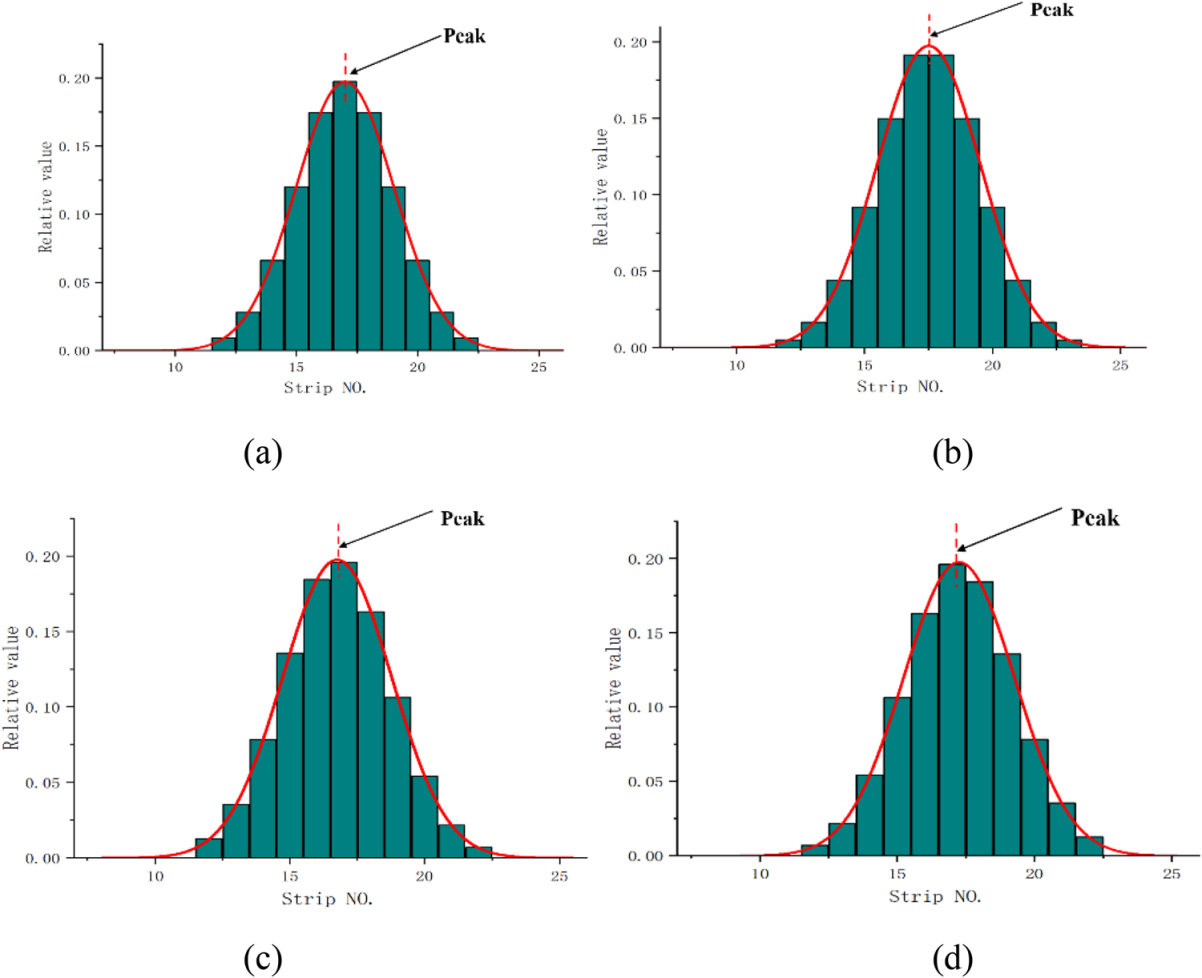
Schematic illustration of the approximation principle (under 4 conditions).

Therefore, a conversion algorithm is proposed to simulate the position signals of the beam on the ionization chamber strips. During the simulation, the beam position information is converted to IC strip output signals. The algorithm uses the beam center position as a reference point and then adopts lookup tables and approximation principles to convert the beam’s center position coordinates into signal values output on different strips, which is actually equivalent to an approximate inverse operation of a Gaussian distribution. The beam center at any position is ultimately divided into four situations based on the proximity principle. The beam center is in the middle of one strip, in the gap between two strips, in the left quartet of one strip, or in the right quarter of one strip, just as shown in Fig. 5a–d. So, theoretically with this method the maximum deviation can be controlled within ± 0.25 mm. It is worth noting that in the actual treatment process, the accuracy of the beam position is generally required to be within ± 0.5 mm to ± 1.5 mm. Therefore, this approach may meet the requirements for offline commissioning.

**Figure 6:**
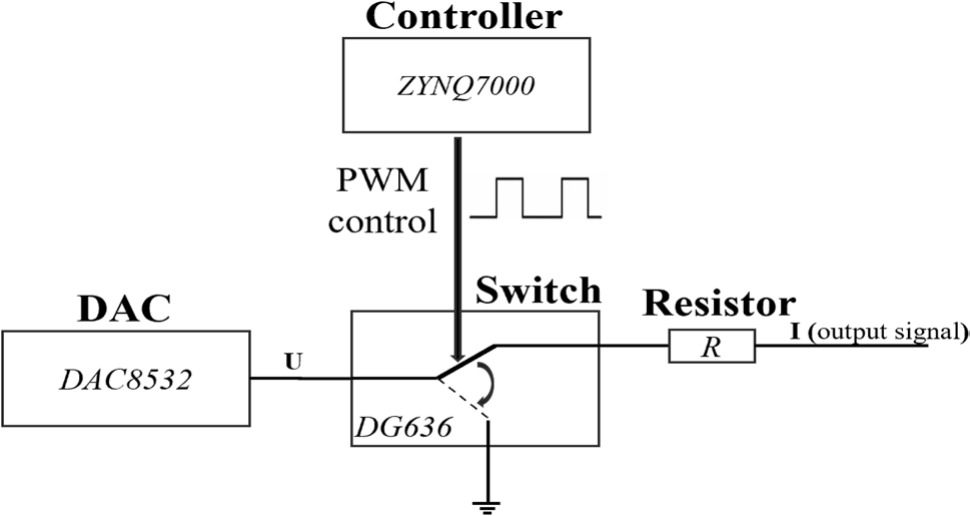
Schematic diagram of a single strip signal generation which includes: the controller, a DAC, a switch, and a resistor.

**Figure 7:**
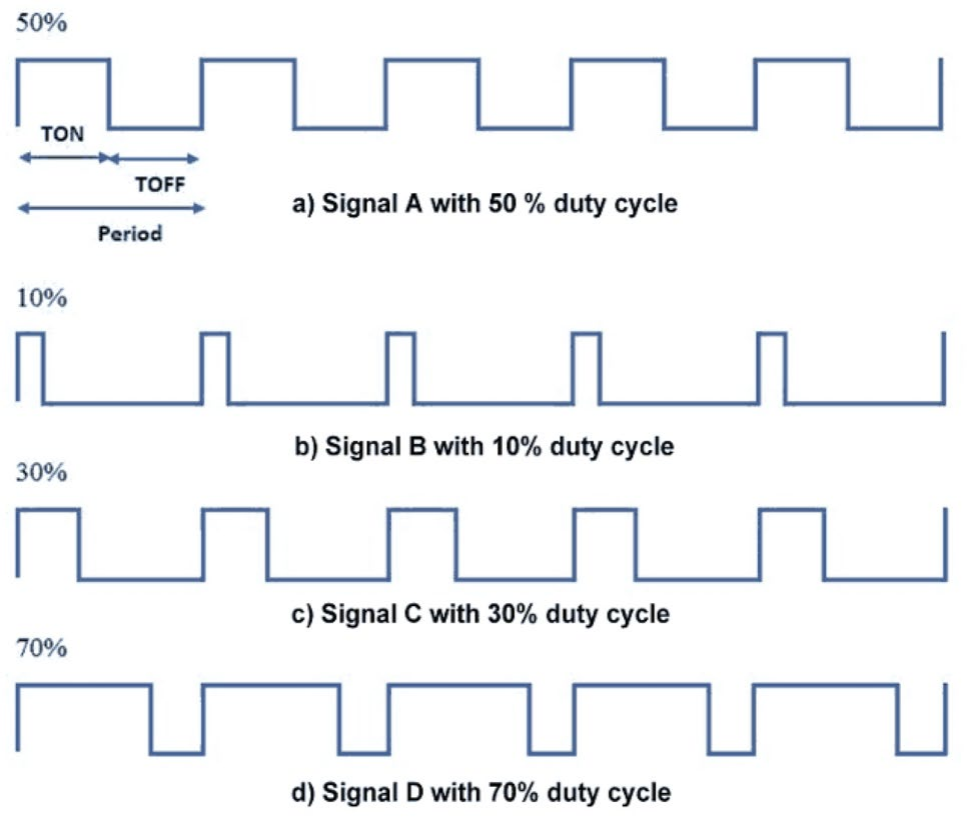
PWM waveforms with different duty cycles.

For the generation of the strip signals, Fig. 6 shows the schematic diagram of a single strip signal generation. The digital to analog converter (DAC) is used to provide a specified voltage. The switch is used to turn on and off the voltage signal, thereby controlling the final output charge. The design principle of the signal output is based on the DAC (DAC8532) plus the high precision low temperature discharge resistor and the fast switch (DG636). The pulse-width modulation (PWM) signal from the core control board can adjust the output signal by controlling the duty cycle, as shown in the formula below:

2 $${\text{Q}}={\text{I}}\times {\text{t}}=\left(\text{U/R}\right)\times {\text{t}},$$

where Q represents the output charge, I denotes the output current, t represents the on-time of PWM wave, U denotes the output voltage of DAC, and R represents the discharge resistance. Figure 7 shows four conditions of PWM waveforms with different duty cycles, and the duty cycle is defined as the ratio of on-time to the period. The duration of the turn-on time increases with increasing duty cycle, resulting in accumulation of the output charge. Consequently, varying output charge can be achieved by controlling the PWM duty cycle (turn-on time). The current (I) during this period can also be obtained. This is only the control of one output signal, and the control of the overall 128 signals can also be achieved by changing the DAC output voltage U. This implies that the overall amplitude of the signals is determined by the beam current, and the relative signals on each strip is determined by the coordinates of the beam center. It should be noted that the beam position information (position and σ) fitted from the overall output signals is the most noteworthy, rather than the value and accuracy of the output signal on each channel.

Since the strip signal is generally very small (tens of nA), the injection charge and leakage current of the switch are required to be as small as possible. Also, the turn-on and turn-off time of the switch should be as short as possible, better under 100 ns. Since the integration time of the IC frontend electronics (composed of 128-channel charge integrating amplifiers and ADC) is usually 100 μs or longer, thus the PWM period should be set no more than 10 μs, at least one tenth of the integration time to ensure the integrity of the integration process and the accuracy and smoothness of the read signals. Consequently, the DG636 chip is selected as the switch, with the leakage current less than 0.5nA, and the turn-on/turn-off time less than 80 ns^24^.

Lookup tables corresponding to the four situations in Fig. 4 are presented as Tables 1, 2, 3 and 4 (σ = 4, 100 MeV), where the default signal values generated on the other strips is zero. The duty cycle of the strip signal is determined based on the percentage of the peak strip, which is set as 100%. Considering the strip width and beam spot size, according to the 3 Sigma rule, 13 strip signals, a total range of 26 mm, are sufficient to simulate the beam signals.

**Table 1:** Strip signals distribution (peak at strip NO.n center).

Strip NO.	Fraction of the beam passing through	(Duty cycle) percentage to max value
n − 6	0.0024	1.2
n − 5	0.0092	4.7
n − 4	0.028	14.2
n − 3	0.066	33.4
n − 2	0.12	60.8
n − 1	0.17467	88.5
n	0.19741	100
n + 1	0.17467	88.5
n + 2	0.12	60.8
n + 3	0.066	33.4
n + 4	0.028	14.2
n + 5	0.0092	4.7
n + 6	0.0024	1.2

**Table 2:** Strip signals distribution (Peak at gap center, strip NO.n − 1 and NO.n).

Strip NO.	Fraction of the beam passing through	Percentage to max value (duty cycle)
n − 6	0.0049	2.6
n − 5	0.0165	8.6
n − 4	0.0441	23.0
n − 3	0.0918	47.9
n − 2	0.1499	78.3
n − 1	0.1915	100
n	0.1915	100
n + 1	0.1499	78.3
n + 2	0.0918	47.9
n + 3	0.0441	23.0
n + 4	0.0165	8.6
n + 5	0.0049	2.6
n + 6	0.0011	0.6

**Table 3:** Strip signals distribution (peak at left quarter of strip NO.n).

Strip NO.	Fraction of the beam passing through	Percentage to max value (duty cycle)
n − 6	0.0034	1.7
n − 5	0.0125	6.4
n − 4	0.0353	18.0
n − 3	0.0782	39.9
n − 2	0.1357	69.3
n − 1	0.1843	94.1
**n**	0.1959	100
n + 1	0.163	83.2
n + 2	0.1062	54.2
n + 3	0.0542	27.7
n + 4	0.0216	11.0
n + 5	0.0068	3.5
n + 6	0.0017	0.9

**Table 4:** Strip signals distribution (d. Peak at right quarter of strip NO.n).

Strip NO.	Fraction of the beam passing through	Percentage to max value (duty cycle)
n − 6	0.0017	0.9
n − 5	0.0068	3.5
n − 4	0.0216	11.0
n − 3	0.0542	27.7
n − 2	0.1062	54.2
n − 1	0.163	83.2
**n**	0.1959	100
n + 1	0.1843	94.1
n + 2	0.1357	69.3
n + 3	0.0782	39.9
n + 4	0.0353	18.0
n + 5	0.0125	6.4
n + 6	0.0034	1.7

As for the amplitude of the beam fluence output signal and the overall tendency of the strip signals, the gain of the ionization chamber and the beam signal itself determine the size of the output signal. As shown in Formula (3), the gain (G) is defined as the output current of the ion chamber (I_C_) divided by the beam current (I_B_), which also determines the output value of DAC. Table 5 shows the typical IC gain (per millimeter gap), the beam size (sigma), and the range in the water of the proton beam, with which the amplitude and range of the IC signals can be derived. Since the air gaps of the strip layer and the Int plane are 7 mm and 10 mm (2 × 5 mm), respectively, as shown in Fig. 1, then the IC gain (both the strip layer and the Int plane) can eventually be obtained by multiplying the gap value (7 mm and 10 mm respective) by the per-millimeter-air-gap IC gain, as shown in the second column of Table 5.

**Table 5:** Typical IC gain, sigma, and range in water of the proton beam.

Proton energy (MeV)	Typical IC gain (mm^−1^ air gap)	Sigma (mm)	Range in water (cm)
70	30.8	4.41	4.075
90	25.2	4.18	6.389
100	23.5	4	7.707
120	20.5	3.79	10.65
150	17.5	3.53	15.76
180	15.4	3.38	21.63
200	14.4	3.31	25.93
230	13.5	3.28	32.91

3 $$\text{G}=\frac{{I}_{c}}{{I}_{B}}.$$

## Design of the hardware circuit

**Figure 8:**
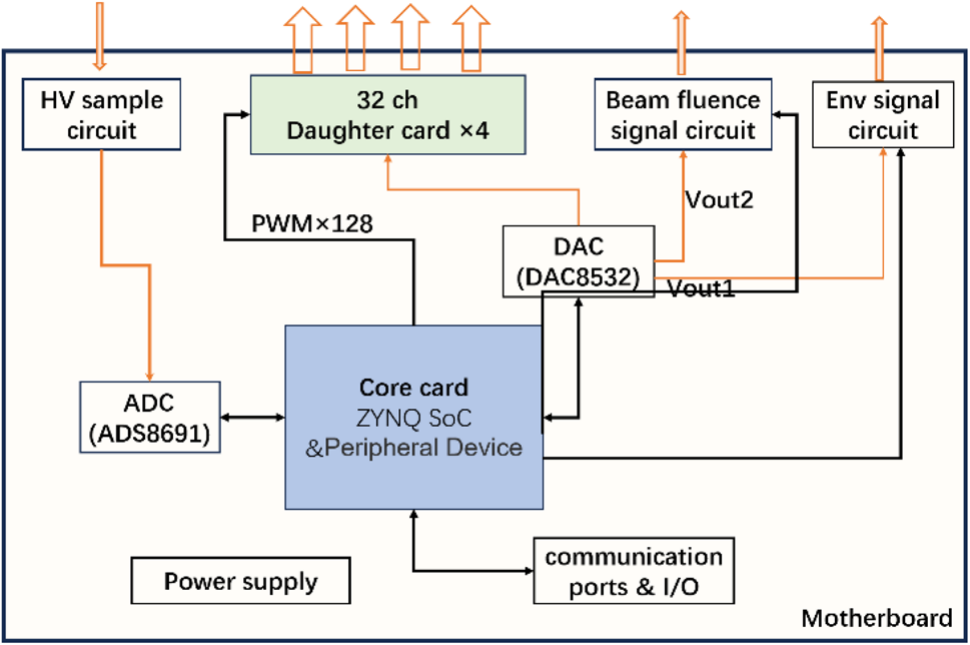
Schematic diagram of hardware architecture.

**Figure 9:**
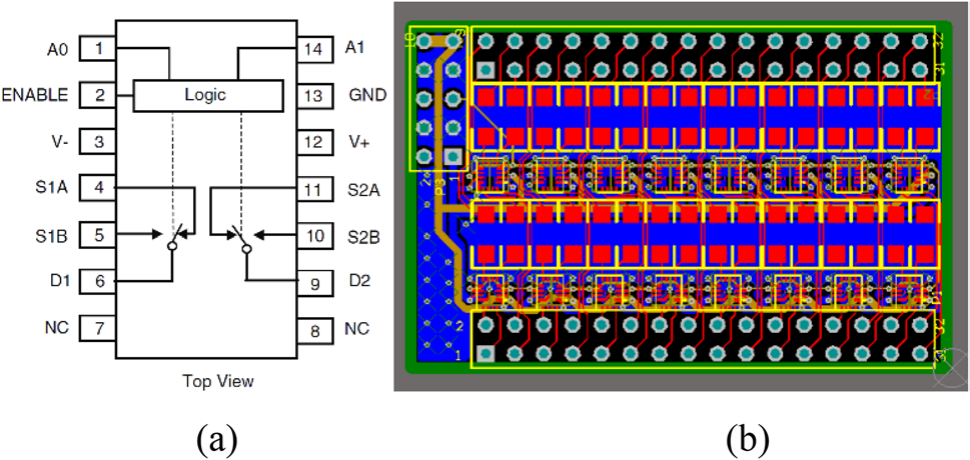
(**a**) DG636 and (**b**) the daughter card PCB.

Figure 8 shows the schematic diagram of hardware architecture. The hardware device consists of three parts: the motherboard, the core card, and the 32-channel daughter card. The motherboard integrates with ADCs and DACs, as well as power supply circuits and communication ports. The beam fluence signal circuit, with a structure of DAC-switch-resistor shown in Fig. 6, can generate the dose message representing the Int plane of the ion chamber. Also, the HV sample circuit is used to check the high voltage from the ion chamber and the Env signal circuit is used to provide the environmental signals (temperature and pressure, 0–10 V voltage) to the ion chamber for calibration. The core card is developed based on the Xilinx SoC, which also integrates with DDR (Double Data Rate SDRAM), flash memory, and other related peripheral circuits. The function of DDR is to serve as an external memory to provide large-capacity and high-bandwidth data storage and reading capabilities for FPGA. Flash is the non-volatile memory that can maintain data even after the device is powered off, also it is cost-effective. So, it is used to save configuration files and store the programmed code. The core card is connected to the motherboard through three high-speed connectors (120Pin × 2, 100Pin × 1). Four daughter cards are used to generate a total of 128 channel signals. Each contains 32 channel signal output circuits, as shown in Fig. 6. Figure 9a shows the schematic package of DG636 and Fig. 9b shows the daughter card PCB.

**Figure 10:**
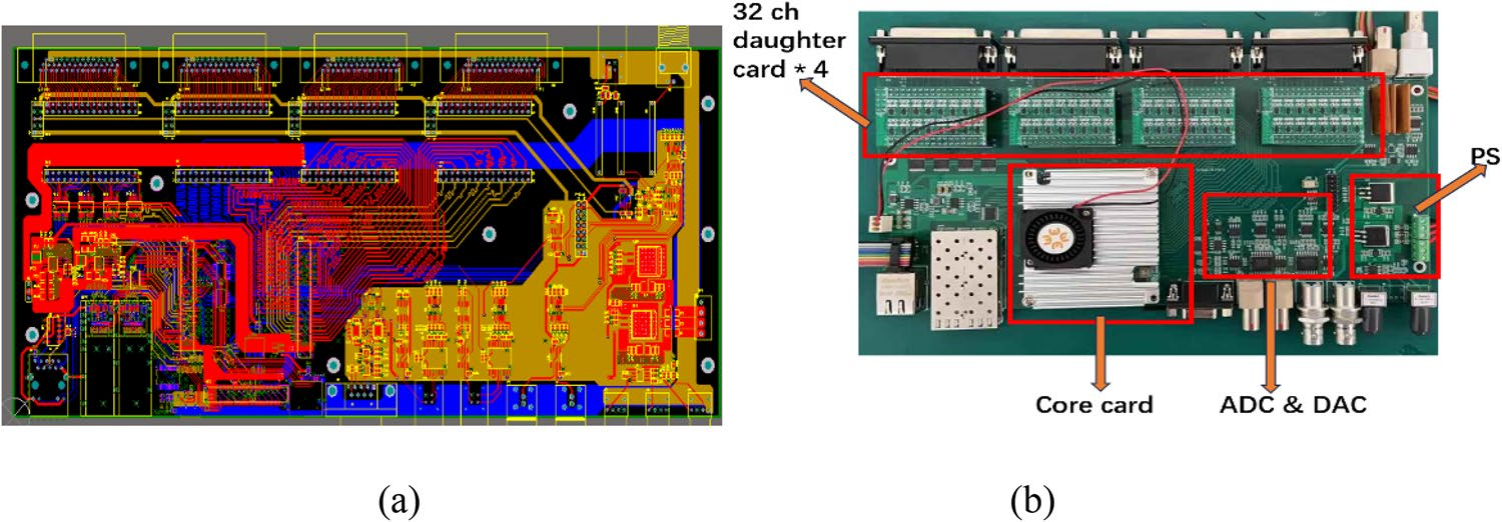
(**a**) PCB of motherboard and (**b**) hardware of the electronics.

Figure 10 shows the PCB and hardware board of the electronics. For the PCB part, the layout, wiring, and grounding treatment are very important. The XILINX SoC (ZYNQ XC7Z035)^25–28^ is selected as the core control unit, which consists of the Processing System (PS) part and the Programmable Logic (PL) part. The PS part handles tasks such as communication with the host computer, data reception and transmission, and data preprocessing. The PL component mainly performs real-time control, such as the PWM signals output and the ADC/DAC driver control. ADAS8691 and DAC8532 are selected as ADC and DAC, respectively, to achieve conversion between the analog and digital quantities. Four 44-pin connectors are used to output the strip signals, and one LEMO connector is used to realize the beam fluence signal output.

## Experimental validation and results analysis

**Figure 11:**
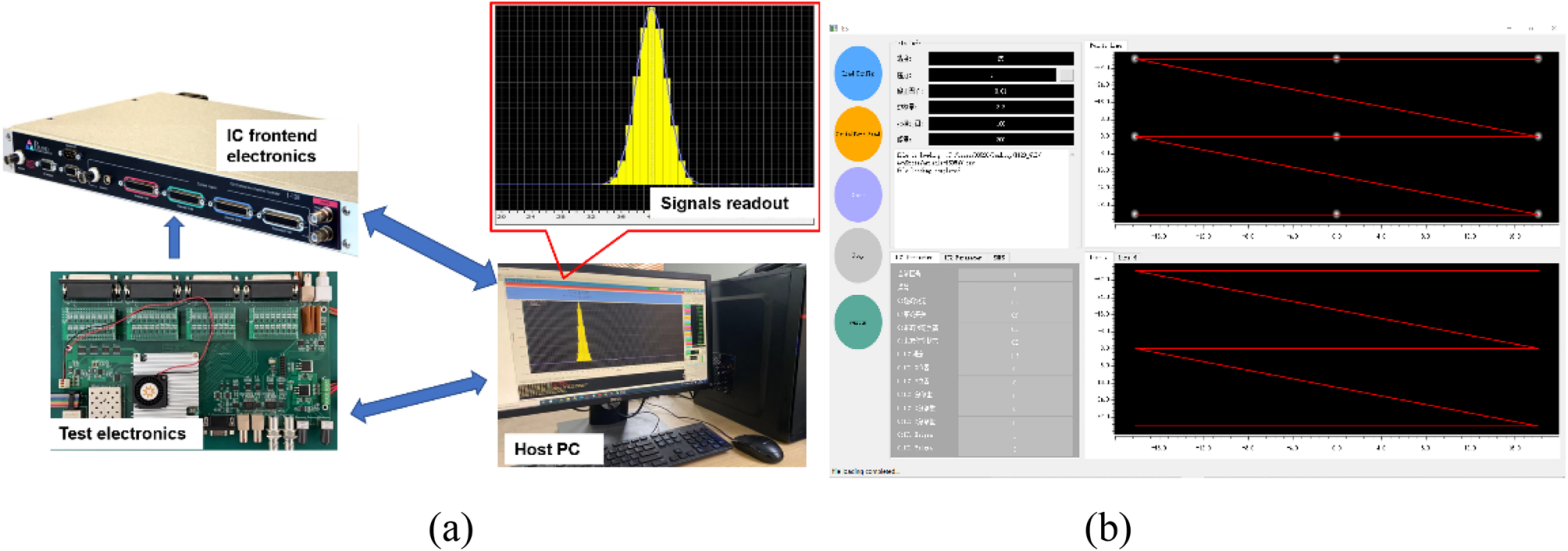
(**a**) The testing system which comprises: the test electronics, the front electronics and the host PC and (**b**) GUI of the test electronics.

The testing system consists of three parts: the test electronics, the IC front-end electronics, and the host PC, as shown in Fig. 11a. In the actual irradiation treatment process, the role of the front-end electronics is to receive signals from the ionization chamber and calculate the beam position as well as the radiation dose. While in the test system, analog signals from the offline-test electronics are sent to the front-end electronics. On the one hand, the host PC sends information related to the irradiation process such as the target position and dose to the test electronics, and on the other hand, it reads data, such as the measured beam position and dose, from the IC front-end electronics. The information mainly includes the target position (x, y), the beam size σ, the target irradiation dose D, the beam current I, and beam energy E, etc. The GUI of the test electronics is illustrated in Fig. 11b.

**Figure 12:**
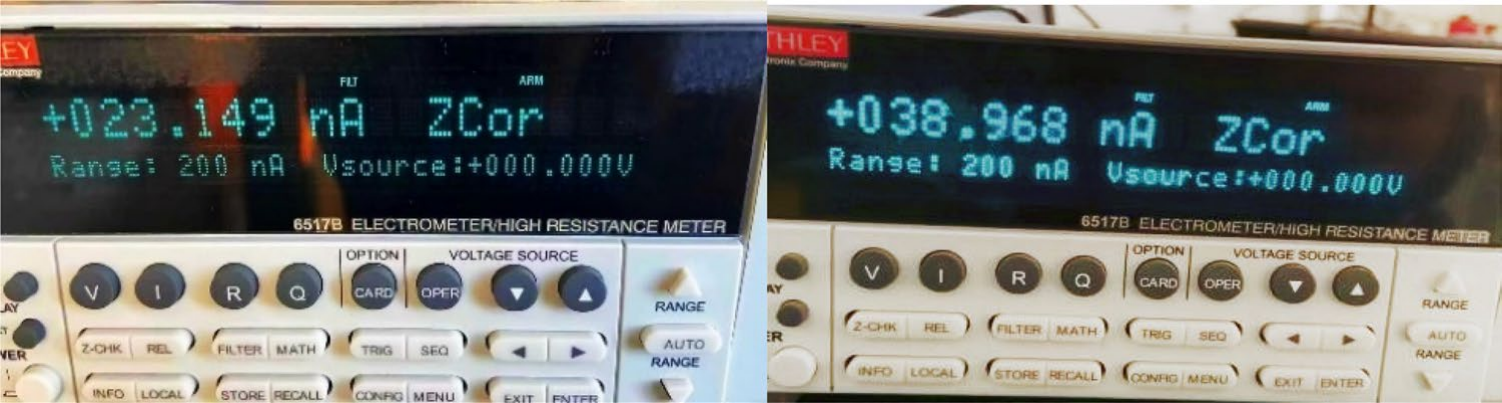
Measurement results of a single output signal.

**Figure 13:**
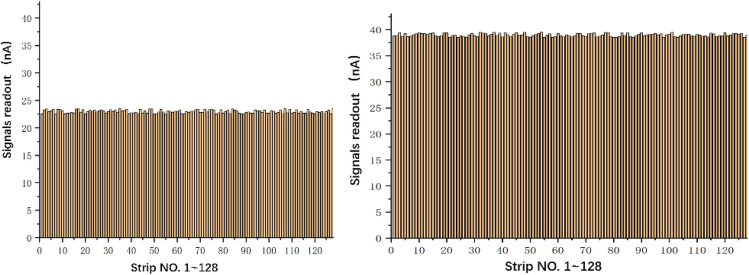
Output of 128 channels.

Firstly, the output of 128 analog signals is tested and the current is measured with Keithley 6517B. Since the gain of the IC strip is approximately from 94 to 216 (gap value multiply per-mm-gap gain, as shown in Table 5), thus assuming the beam current is 1nA, the total signal generated by the strip layer ranges from 94 to 216 nA. The signal on a single strip generally does not exceed 20–30% of the total signal. Therefore, four sets of data were obtained: 23 nA, 39 nA, 50 nA, and 80 nA. The first two sets of data are for the common situations and the last two are for extreme situations. As shown in Fig. 12, one of the channels (e.g. ch20) is randomly selected to be measured, with the read current of 23.149 and 38.968 nA. At the same time, the measurement results of the 128 channels are shown in Fig. 13. For the target output value of 80 nA, the 128 signals were measured to be between 79.6 and 80.5 nA. For the target value of 50 nA, the 128 signals were measured to be between 49.4 and 50.7 nA. The differences are mainly related to the accuracy of the selected resistors and the circuit differences of each channel, but the results indicate that the errors are within ± 1 nA. Furthermore, just as mentioned above, the experimental results are more concerned with the final position data fitted based on the overall 128 signals, rather than the characteristics of a single simulated strip signal.

**Figure 14:**
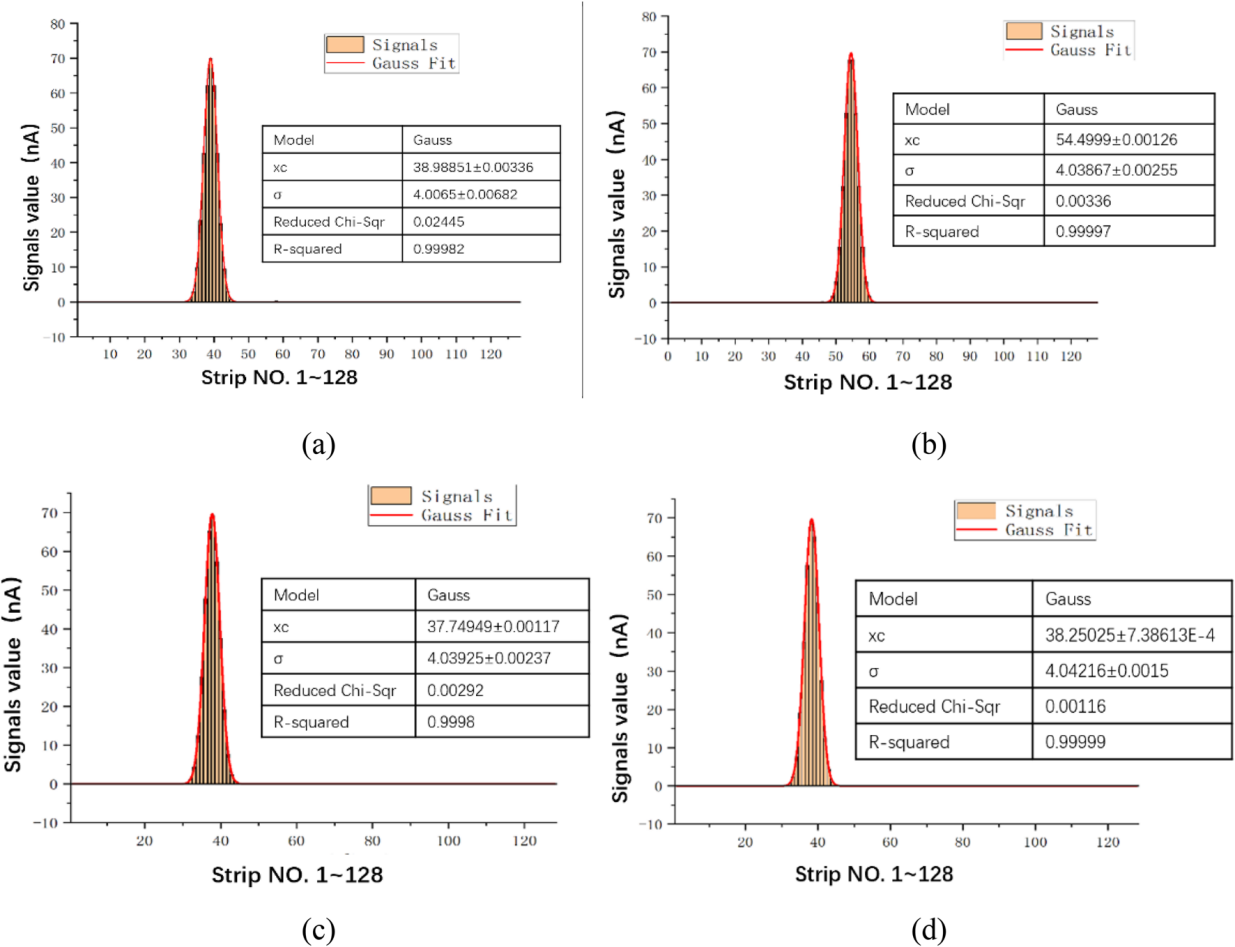
Gaussian signals and fitting results (σ = 4).

Subsequently, the simulated Gaussian distribution signals are tested. The host PC sends radiation-related data to the offline testing electronics, which then outputs the corresponding analog signals to the front-end electronics of the ionization chamber. Finally, the host PC reads the data back from the front-end electronics. The simulated Gaussian signals and the fitting results are shown in Fig. 14 where (a)–(d) correspond to the four situations in Fig. 5. The results, tested under sigma equals 4, show that the output signals conform to a Gaussian distribution accurately, with the R-square value not less than 0.999.

Furthermore, the following formulas are used to convert the fitted strip electrode coordinates into the beam position. These formulas are also used by the host PC to convert the beam position to the strip position, then sent to the test electronics.

4 $$x=\left(Sx-64.5\right)\times S,$$

5 $$y=\left(Sy-64.5\right)\times S.$$

**Figure 15:**
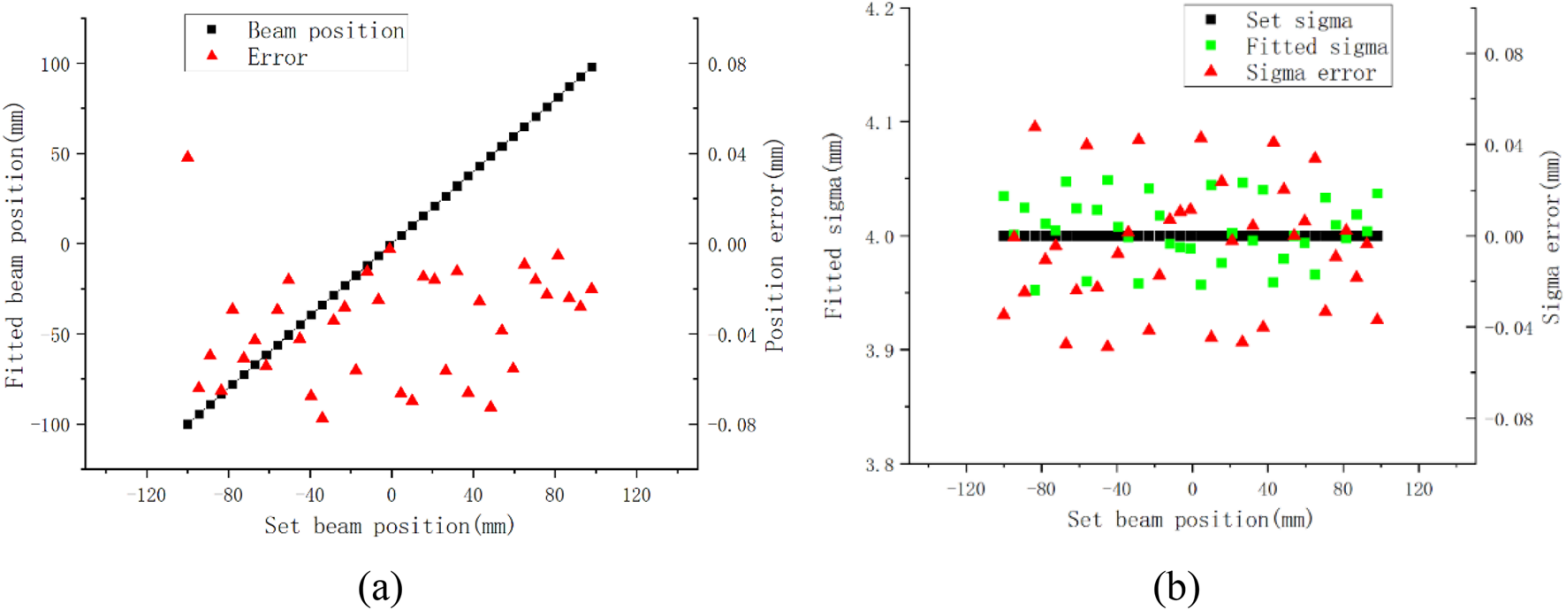
Position errors and sigma errors (σ = 4).

In these equations, (x, y) represent the actual position of the beam. (Sx, Sy) denote the strip electrode coordinates fitted based on the simulated signals, and S represents the width of the strip spacing, which is 2 mm. Then, according to the same test conditions, the position error experiment was carried out every 4.5 mm interval from − 100 to 100 mm. The fitted position error was within ± 0.067 mm, and the sigma error was within ± 0.043 mm, as shown in Fig. 15.

On this basis, multiple repeated experiments were conducted at different energies (70 MeV, 100 MeV, 180 MeV, and 230 MeV), and the results demonstrated that the fitted beam center position errors were all within ± 0.08 mm, with a maximum sigma error of ± 0.05 mm. Combined with the maximum theoretical error of ± 0.25 mm, the final position error can be ensured to be within ± 0.33 mm, which can fully meet the requirements of general testing occasions.

The output of the beam fluence simulation signal and environmental simulation signal is relatively easy and simple. For the beam fluence simulation signals, since the gain of the IC Int plane is generally between 135 and 308 (70 to 230 MeV, calculated by multiplying the data in the second column of Table 5 by the 10mm gap), the output analog current is also in the order of 100 nA. So, six experiments were conducted covering the range from 100 to 350 nA (specifically 100/150/200/250/300/350 nA), and the test results indicated that the accuracy of the simulated beam fluence signals can be easily maintained within ± 1%. Table 6 shows the comprehensive test results with different beam energies.

**Table 6:** Comprehensive test results under various beam energies.

Beam energy (MeV)	Item			
	Beam position error (fitted + theoretical error) (mm)	Beam sigma error (mm)	R-square	Beam fluence signal output error (%)
70	± (0.069 + 0.25)	± 0.043	> 0.999	± 1
100	± (0.067 + 0.25)	± 0.043		
180	± (0.072 + 0.25)	± 0.046		
230	± (0.077 + 0.25)	± 0.049		
70–230	± 0.33	± 0.05	> 0.999	± 1

## Conclusion

In this article, we report the test electronics for outputting 128-channel analog signals to simulate the beam position information, and other signals such as the beam fluence signal and the environmental signals are also generated to feed to the IC front-end electronics. With the test electronics, it is possible to test and upgrade the nozzle system and the entire treatment device offline (no proton beam or the accelerator) at any time, without considering the beam quality or the on-site dose after the experiment. It provides a very convenient, rapid, and reliable way for the commissioning of the proton therapy facility, and provides a potential avenue for operator training. The offline test electronics has successfully solved the key problem of generating simulated signals related to the beam, laying a robust foundation for the overall offline test system including the accelerator and the beamline in the future.

## Data Availability

The datasets used and/or analysed during the current study available from the corresponding author on reasonable request.

## References

[1] Zhang T, Wang C, Li M (2017). Developments for 230 MeV superconducting cyclotrons for proton therapy and proton irradiation. Nucl. Instrum. Methods Phys. Res..

[2] Zhang T, Lin J, Yin Z (2017). Design and construction of the main magnet for a 230-MeV superconducting cyclotron. ITAS.

[3] Zhang T, Li M, Wang C (2020). Investigation and quantitative simulation of beam physics for 230 MeV SC cyclotron under construction at CIAE. Nucl. Instrum. Methods Phys. Res. Sect. B.

[4] Yin Z, Ji B, Fu X (2017). Design of RF system for Cyciae-230 superconducting cyclotron. Nucl. Instrum. Methods Phys. Res..

[5] Kawachi K, Kanai T, Matsuzawa H (1983). Three dimensional spot beam scanning method for proton conformation radiation therapy. Acta Radiol. Suppl..

[6] Schippers, J. M., Meer, D. & Pedroni E. Fast-scanning techniques for cancer therapy with hadrons, a domain of cyclotrons. In *Proc. 19th International Conference on Cyclotrons and Their Applications, Lanzhou, China* 410–415 (2010).

[7] Marchand, B., Prieels, D., Bauvir, B., Sépulchre, R. & Gérard, M. IBA proton pencil beam scanning: An innovative solution for cancer treatment. In *Proc. EPAC 2000* 2539–2541.

[8] Klimpki G, Eichin M, Bula C (2018). Real-time beam monitoring in scanned proton therapy. Nucl. Instrum. Methods Phys. Res. Sect..

[9] Furukawa (2010). Performance of the NIRS fast scanning system for heavy-ion radiotherapy. Med. Phys..

[10] Schippers JM (2009). Beam delivery systems for particle radiation therapy: Current status and recent developments. Rev. Acceler. Sci. Technol..

[11] Peng H, Yin Z-G, Mu X-E (2021). Design of the front-end electronics for parallel plate ion chamber. Radiat. Detect. Technol. Methods.

[12] https://ptcusa.com/products/ic128-25.

[13] Hofverberg P, Bergerot JM, Trimaud R (2021). The development of a treatment control system for a passive scattering proton therapy installation. Nucl. Instrum. Methods Phys. Res. Sect. A.

[14] Smith A, Gillin M, Bues M (2009). The MD Anderson proton therapy system. Med. Phys..

[15] Zhu XR, Poenisch F, Lii M, Sawakuchi GO, Titt U, Bues M, Song X, Zhang X, Li Y, Ciangaru G, Li H (2013). Commissioning dose computation models for spot scanning proton beams in water for a commercially available treatment planning system. Med. Phys..

[16] Gillin MT, Sahoo N, Bues M (2009). Commissioning of the discrete spot scanning proton beam delivery system at the University of Texas MD Anderson Cancer Center, Proton Therapy Center, Houston. Med. Phys..

[17] Safai S, Bula C, Meer D (2012). Improving the precision and performance of proton pencil beam scanning. Transl. Cancer Res..

[18] Pidikiti R, Patel BC, Maynard MR (2018). Commissioning of the world’s first compact pencil-beam scanning proton therapy system. J. Appl. Clin. Med. Phys..

[19] Giordanengo, S, & Donetti, M. *Dose Delivery Concept and Instrumentation*. 10.23730/CYRSP-2017-001.13 (2018).

[20] Saini J, Cao N, Bowen SR (2016). Clinical commissioning of a pencil beam scanning treatment planning system for proton therapy. Int. J. Part. Ther..

[21] Kim J (2003). Proton therapy facility project in National cancer center, Korea. J. Korean Phys. Soc..

[22] Scisciò M, Migliorati M, Palumbo L (2018). Design and optimization of a compact laser-driven proton beamline. Sci. Rep..

[23] Newhauser WD, Zhang R (2015). The physics of proton therapy. Phys. Med. Biol..

[24] https://www.digikey.cn/zh/products/detail/vishay-siliconix/DG636EEN-T1-GE4/7422973.

[25] Vlagkoulis V (2021). Single event effects characterization of the programmable logic of xilinx Zynq-7000 FPGA using very/ultra high-energy heavy ions. IEEE Trans. Nucl. Sci..

[26] Amrbar, M., Irom, F., Guertin, S. M. & Allen, G. Heavy ion single event effects measurements of xilinx Zynq-7000 FPGA. In *2015 IEEE Radiation Effects Data Workshop (REDW), Boston, MA, USA* 1–4. 10.1109/REDW.2015.7336714 (2015).

[27] Ahmad S, Boppana V, Ganusov I, Kathail V, Rajagopalan V, Wittig R (2016). A 16-nm multiprocessing system-on-chip field-programmable gate array platform. IEEE Micro.

[28] https://china.xilinx.com/products/silicon-devices/soc/zynq-7000.html#productTable.

